# Antimicrobial activity of omadacycline in vitro against bacteria isolated from 2014 to 2017 in China, a multi-center study

**DOI:** 10.1186/s12866-020-02019-8

**Published:** 2020-11-16

**Authors:** Meng Xiao, Jing-jing Huang, Ge Zhang, Wen-hang Yang, Fanrong Kong, Timothy Kudinha, Ying-chun Xu

**Affiliations:** 1 Department of Clinical Laboratory, Peking Union Medical College Hospital, Peking Union Medical College, Chinese Academy of Medical Sciences, Beijing, 100730 China; 2 Beijing Key Laboratory for Mechanisms Research and Precision Diagnosis of Invasive Fungal Diseases, Beijing, 100730 China; 3 Graduate School, Peking Union Medical College, Chinese academy of Medical Science, Beijing, 100730 China; 4 Centre for Infectious Diseases and Microbiology Laboratory Services, ICPMR-Pathology West, Westmead Hospital, Westmead, NSW Australia; 5 Charles Sturt University, Leeds Parade, Orange, Sydney, NSW Australia; 6 NSW Health Pathology, Regional and Rural, Orange Hospital, Orange, NSW Australia

**Keywords:** Omadacycline, Aminomethylcycline, Tetracyclines, *Streptococcus pneumoniae*, *Staphylococcus aureus*

## Abstract

**Background:**

Omadacycline (ZL-2401) is a semi-synthetic derivative of minocycline. It has a broadspectrum activity against Gram-positive and Gram-negative bacteria, and atypical pathogens. The objective of this study was to evaluate the antibacterial activity of omadacycline against recently collected bacterial isolates from Chinese patients.

**Results:**

Omadacycline showed potent activity against all Gram-positive pathogens: *S. aureus* MICs were low regardless of susceptibility to methicillin (methicillin-resistant *Staphylococcus aureus*, MRSA: *N* = 97, MIC_50/90_ 0.12/0.25 mg/L, 98.5% susceptible; methicillin-sensitive *Staphylococcus aureus*, MSSA: *N* = 100, MIC_50/90_ 0.12/0.12 mg/L, 100.0% susceptible). Omadacycline was also very effective against β-haemolytic streptococci (MIC_50/90_, 0.06/0.12 mg/L), viridans group streptococci (MIC_50/90_,<0.03/0. 06 mg/L), and enterococci (MIC_50/90_, 0.03/0.12 mg/L). Against *S. pneumoniae*, omadacycline was highly active regardless of penicillin-resistance (MIC_90_ 0.06 mg/L) and despite the fact that less than 10.0% of these strains were susceptible to tetracycline. Omadacycline exhibited good in vitro activity against *Enterobacterales* isolates (MIC_50/90_, 2/8 mg/L), inhibiting 81.7% of the isolates at ≤4 mg/L. *M. catarrhalis* isolates (MIC_50/90_, 0.12/0.25 mg/L) were fully susceptible to omadacycline at ≤0.5 mg/L.

**Conclusions:**

Omadacycline showed potent in vitro activity against most common bacterial pathogens, and even against highly resistant problem pathogens, such as MRSA, penicillin-R and tetracycline-R *S. pneumoniae* and enterococci. The susceptibility rate of Chinese isolates was similar to those reported in other countries, but the decreased activity against *K. pneumoniae* isolates in the present study should be noted.

## Background

The main bacterial pathogens of acute bacterial skin and skin structure infections (ABSSSIs) include *Staphylococcus aureus* and coagulase-negative *staphylococci*, with the former exhibiting high antimicrobial resistance rate and thus requiring highly effective antibiotics for management [1]. Community-acquired bacterial pneumonia (CABP) poses a significant health and economic burden in all regions of the world [1]. The most prevalent pathogens implicated in CABP include *Streptococcus pneumoniae*, *Haemophilus influenzae*, *Moraxella catarrhalis*, *Mycoplasma pneumoniae*, *Chlamydophila pneumoniae*, *Klebsiella pneumoniae*, and *Pseudomonas aeruginosa* [2, 3]. Among them, penicillin-resistant and multidrug-resistant *S. pneumoniae* (PRSP) are particularly concerning.

The tetracycline family of antibiotics entered the clinical practice against common infectious diseases in the late 1940s. However, due to the excessive use of antimicrobials in agriculture and veterinary medicine for many years, the resistance rates of several bacterial strains in environmental and animal reservoirs have increased to worrying levels, including the emergence of tetracycline resistant strains [4]. In the past decade, tigecycline has replaced tetracycline as an important therapeutic drug for antimicrobial resistant bacterial strains [5]. However, tigecycline is unavailable in oral form and has been reported to be associated with a high incidence of nausea and vomiting, and is even implicated in elevated all-cause mortality [6, 7].

Omadacycline (ZL-2401) is a novel antibacterial agent approved by the US Food and Drug Administration (FDA) for the treatment of ABSSSIs and CABP [7, 8]. As a semi-synthetic derivative of minocycline, and the first agent of the aminomethylcycline class, it has a broad spectrum activity against a wide range of organisms, including Gram-positive and Gram-negative bacteria [9]. Even against highly resistant bacterial strains such as methicillin-resistant *S. aureus* (MRSA), vancomycin-resistant enterococci (VRE), and PRSP, good antimicrobial activity has also been observed as per data obtained in the SENTRY antimicrobial surveillance program [10]. However, the prevalence and antimicrobial resistance rates of some bacterial species are different all over the world [11]. The aim of this study was to evaluate the in vitro activity of omadacycline against common bacteria in Mainland China.

## Results

### Susceptibility of gram-positive isolates to omadacycline

The 1273 bacterial isolates studied and their key resistance phenotypes are listed in Tables 1 and 2. The MIC distributions of Gram-positive isolates against omadacycline are shown in Tables 1 and 3. Omadacycline was tested against *S. aureus* isolates (98.5% susceptible), using ABSSSI breakpoints with MIC_50/90_ values of 0.12/0.25 mg/L (Table 1). Of these, 100.0% of MSSA, 96.9% of MRSA, 97.4% of tetracycline-resistant *S. aureus*, and 100.0% of tigecycline-resistant *S. aureus*, were susceptible to omadacycline.

**Table 1:** Antimicrobial activity of omadacycline against Gram-positive cocci organisms

Organism/organism group (number of isolates)	Number (cumulative %) of isolates at MIC (mg/L) of:								MIC_50_	MIC_90_	%S	%R
	<0.03	0.03	0.06	0.12	0.25	0.5	1	2				
*Staphylococcus aureus*(197)	0 (0.0)	1 (0.5)	43 (22.3)	118 (82.2)	27 (95.9)	5 (98.5)	2 (99.5)	1 (100.0)	0.12	0.25	98.5^a^	0.5
Methicillin-susceptible (100)	0 (0.0)	1 (1.0)	27 (28.0)	69 (97.0)	2 (99.0)	1 (100.0)			0.12	0.12	100.0	0.0
Methicillin-resistant (97)			16 (16.5)	49 (67.0)	25 (92.8)	4 (96.9)	2 (99.0)	1 (100.0)	0.12	0.25	96.9	1.0
Tetracycline-resistant (116)	0 (0.0)	1 (0.9)	19 (17.2)	67 (75.0)	21 (93.1)	5 (97.4)	2 (99.1)	1 (100.0)	0.12	0.25	97.4	0.9
Tigecycline-non-susceptible (1)			0 (0.0)	1 (100.0)							100.0	0.0
*Enterococcus* spp. (25)	0 (0.0)	13 (52.0)	7 (80.0)	5 (100.0)					0.03	0.12		
*Enterococcus faecalis* (9)	0 (0.0)	2 (22.2)	4 (66.7)	9 (100.0)					0.06		100.0	0.0
*Enterococcus faecium* (16)	0 (0.0)	11 (68.8)	3 (87.5)	2 (100.0)					0.03	0.12		
*Streptococcus pneumoniae* (59)	6 (10.2)	21 (45.8)	27 (91.5)	3 (96.6)	2 (100.0)				0.06	0.06	96.6	0.0
Penicillin-susceptible (25)	3 (12.0)	8 (44.0)	11 (88.0)	1 (92.0)	2 (100.0)				0.06	0.12	92.0	0.0
Penicillin-intermediate (15)	1 (6.7)	6 (46.7)	7 (93.3)	1 (100.0)					0.06	0.06	100.0	0.0
Penicillin-resistant (19)	2 (10.5)	7 (47.3)	9 (94.7)	1 (100.0)					0.06	0.06	100.0	0.0
Tetracycline-resistant (53)	4 (7.5)	18 (41.5)	26 (90.6)	3 (96.2)	2 (100.0)				0.06	0.06	96.2	0.0
Tigecycline-non-susceptible (3)	0 (0.0)	1 (33.3)	0 (33.3)	0 (33.3)	2 (100.0)				0.25		33.3	0.0
Viridans group streptococci (25)	14 (56)	8 (88.0)	1 (92.0)	1 (96.0)	1 (100.0)				0.015	0.06		
*Streptococcus anginosus* group (21)	11 (52.4	7 (85.7)	1 (90.5)	1 (95.2)	1 (100.0)				0.015	0.06	95.2	0.0
Tetracycline-resistant (14)	5 (35.7)	7 (85.7)	1 (92.9)	1 (100.0)					0.03	0.06	100.0	0.0
Tigecycline-non-susceptible (1)				0 (0.0)	1 (100.0)						0.0	0.0
β-haemolytic streptococci (27)	1 (3.7)	11 (44.4)	6 (66.7)	8 (96.3)	1 (100.0)				0.06	0.12		
Tetracycline-resistant (20)	1 (5.0)	4 (25.0)	6 (55.0)	8 (95.0)	1 (100.0)				0.06	0.12		
*Streptococcus pyogenes* (8)	1 (12.5)	7 (100.0)							0.03		100.0	0.0

^a^ Applying FDA identified breakpoints for ABSSSIs

**Table 2:** Antimicrobial activity of omadacycline against Gram-negative bacilli organisms

Organism/organism group (number of isolates)	Number (cumulative %) of isolates at MIC (mg/L) of:										MIC_50_	MIC_90_	%S	%R
	≤0.06	0.12	0.25	0.5	1	2	4	8	16	32				
*Enterobacterales* (651)	1 (0.2)	0 (0.2)	11 (1.8)	86 (15.1)	182 (43.0)	151 (66.2)	101 (81.7)	63 (91.4)	31 (96.2)	25 (100.0)	2	8		
Ceftazidime-non-susceptible (275)	1 (0.4)	0 (0.4)	5 (2.2)	30 (13.1)	64 (36.4)	59 (57.8)	51 (76.4)	31 (87.6)	15 (93.1)	19 (100.0)	2	16		
Tetracycline-resistant (401)		0 (0.0)	1 (0.2)	40 (10.2)	108 (37.2)	76 (56.1)	66 (72.6)	54 (86.0)	31 (93.8)	25 (100.0)	2	16		
Tigecycline-non-susceptible (55)						0 (0.0)	2 (3.6)	12 (25.5)	18 (58.2)	23 (100.0)	16	32		
*Escherichia coli* (260)		0 (0.0)	8 (3.1)	60 (26.2)	107 (67.3)	52 (87.3)	13 (92.3)	8 (95.4)	0 (95.4)	12 (100.0)	1	4		
Carbapenemase-producing (19)			0 (0.0)	6 (31.6)	6 (63.2)	4 (84.2)	3 (100.0)				1	4		
ESBL phenotype positive (134) ^a^		0 (0.0)	6 (4.5)	28 (25.4)	57 (67.9)	16 (79.9)	9 (86.6)	6 (91.0)	0 (91.0)	12 (100.0)	1	8		
ESBL phenotype negative (107) ^a^		0 (0.0)	2 (1.9)	26 (26.2)	44 (67.3)	32 (97.2)	1 (98.1)	2 (100.0)			1	2		
Tetracycline-resistant (221)		0 (0.0)	1 (0.5)	36 (16.7)	99 (61.5)	52 (85.1)	13 (91.0)	8 (94.6)	0 (94.6)	12 (100.0)	1	4		
Tigecycline-non-susceptible (1)									0 (0.0)	1 (100.0)				
*Klebsiella pneumoniae* (271)		0 (0.0)	2 (0.7)	21 (8.5)	54 (28.4)	62 (51.3)	52 (70.5)	44 (86.7)	25 (95.9)	11 (100.0)	2	16	70.5 ^b,c^	13.3 ^b,c^
Carbapenemase-producing (47)			0 (0.0)	3 (6.4)	0 (6.4)	13 (34.0)	18 (72.3)	6 (85.1)	5 (95.7)	2 (100.0)	4	16	72.3 ^b,c^	14.9 ^b,c^
ESBL phenotype positive (118) ^a^			0 (0.0)	4 (3.4)	14 (15.3)	25 (36.4)	25 (57.6)	28 (81.4)	13 (92.4)	9 (100.0)	4	16	57.6 ^b,c^	18.6 ^b,c^
ESBL phenotype negative (106) ^a^		0 (0.0)	2 (1.9)	14 (15.1)	40 (52.8)	24 (75.5)	9 (84.0)	10 (93.4)	7 (100.0)		1	8	84.0 ^b,c^	6.6 ^b,c^
Tetracycline-resistant (127)			0 (0.0)	2 (1.6)	4 (4.7)	16 (17.3)	31 (41.7)	38 (71.7)	25 (91.3)	11 (100.0)	8	16	41.7 ^b,c^	28.3 ^b,c^
Tigecycline-non-susceptible (6)								0 (0.0)	2 (33.3)	4 (100.0)	32		0.0 ^b,c^	100.0^b,c^
*Klebsiella aerogenes* (30)				0 (0.0)	6 (20.0)	13 (63.3)	4 (76.7)	5 (93.3)	2 (100.0)		2	8		
*Enterobacter cloacae* (30)				0 (0.0)	3 (10.0)	16 (63.3)	9 (93.3)	0 (93.3)	0 (93.3)	2 (100.0)	2	4	93.3^b^	6.7 ^b^
Ceftazidime-non-susceptible (16)				0 (0.0)	1 (6.2)	10 (68.8)	4 (93.8)	0 (93.8)	0 (93.8)	1 (100.0)	2	4	93.8^b^	6.2 ^b^
Tetracycline-resistant (11)				0 (0.0)	1 (9.1)	3 (36.4)	5 (81.8)	0 (81.8)	0 (81.8)	2 (100.0)	4	32	81.8^b^	18.2^b^
Citrobacter spp. (30)	1 (3.3)	0 (3.3)	1 (6.7)	5 (23.3)	12 (63.3)	5 (80.0)	2 (86.7)	2 (93.3)	2 (100.0)		1	8		
Tetracycline-resistant (11)			0 (0.0)	2 (18.2)	3 (45.5)	3 (72.7)	1 (81.8)	0 (81.8)	2 (100.0)		2	16		
*Serratia marcescens* (30)					0 (0.0)	3 (10.0)	21 (80.0)	4 (93.3)	2 (100.0)		4	8		
*Acinetobacter baumannii* (198)	8 (4.0)	14 (11.1)	15 (18.7)	7 (22.2)	16 (30.3)	38 (49.5)	52 (75.8)	24 (87.9)	18 (97.0)	6 (100.0)	4	16		
*Pseudomonas aeruginosa* (30)					0 (0.0)	1 (3.3)	0 (3.3)	8 (30.0)	0 (30.0)	21 (100.0)	32	32		
*Stenotrophomonas maltophilia* (30)				0 (0.0)	4 (13.3)	12 (53.3)	11 (90.0)	3 (100.0)			2	4		
*Moraxella catarrhalis* (31)	1 (3.2)	25 (83.9)	5 (100.0)								0.12	0.25		

^a^ *ESBL*: extended-spectrum β-lactamase

^b^ Using FDA identified breakpoints for ABSSSIs

^c^ Using FDA identified breakpoints for CABP

**Table 3:** Activity of omadacycline and comparator agents against a range of bacterial pathogens from 25 teaching hospitals in China

Antimicrobial agent by organism or organism group (no. tested)	MIC_50_ (mg/L)	MIC_90_ (mg/L)	S (%)	R (%)	NS (%)
*Staphylococcus aureus* (197)					
Omadacycline	0.12	0.25	98.5 ^a^	0.5 ^a^	
Tigecycline	0.12	0.25	99.5		0.5
Tetracycline	16	64	36.0	58.9	
Clindamycin	0.12	8	64.5	34.0	
Daptomycin	0.5	1	99.0		1.0
Gentamicin	0.5	64	59.9	33.5	
Levofloxacin	4	8	48.2	50.8	
Linezolid	1	2	99.5	0.5	
Oxacillin	2	8	50.8	49.2	
Trimethoprim-sulfamethoxazole	0.25	1	96.4	3.6	
Vancomycin	1	1	100.0	0.0	
*Enterococcus* spp. (25)					
Omadacycline	0.03	0.12	100.0 ^b^	0.0 ^b^	
Tigecycline	0.12	0.25	100.0		0.0
Minocycline	16	16	40.0	52.0	
Tetracycline	64	64	24.0	76.0	
Daptomycin	1	2	100.0	0.0	
Moxifloxacin	8	8	16.0	84.0	
Linezolid	2	2	100.0	0.0	
Vancomycin	1	2	96.0	4.0	
*Streptococcus pneumoniae* (59)					
Omadacycline	0.06	0.06	96.6	0.0	
Tigecycline	<0.03	0.03	94.9		5.1
Tetracycline	32	64	6.8	89.8	
Ceftriaxone	0.25	4	81.4	11.9	
Erythromycin	2	2	22.0	76.3	
Levofloxacin	1	1	98.3	1.7	
Linezolid	1	1	100.0	0.0	
Penicillin	0.12	4	89.8	1.7	
*Enterobacterales* (651)					
Omadacycline	2	8	81.7 ^c^	8.6 ^c^	
Tigecycline ^d^	0.5	2	91.6 ^d^	1.1 ^d^	
Minocycline	4	32	34.6		56.2
Tetracycline	64	64	33.9	61.6	
Amikacin	4	16	85.7	8.6	
Ciprofloxacin	2	64	57.8	35.6	
Ceftazidime	64	64	42.1	56.2	
Ceftriaxone	0.5	2	85.3	8.8	
Imipenem	1	4	40.7	52.7	
Piperacillin-tazobactam	4	128	70.5	24.1	
*Escherichia coli* (260)					
Omadacycline	1	4	92.3	4.6	
Tigecycline ^d^	0.25	1	94.6 ^d^	0.4 ^d^	
Minocycline	8	21	0.0	99.6	
Tetracycline	64	64	14.6	85.0	
Aztreonam	8	32	46.2	49.6	
Amikacin	4	8	80.0	7.7	
Ciprofloxacin	4	4	29.2	63.5	
Ceftazidime	2	64	59.2	32.7	
Ceftriaxone	64	64	41.2	58.5	
Imipenem	0.5	0.5	96.2	2.7	
Piperacillin-tazobactam	4	128	81.9	13.5	
*Klebsiella pneumoniae* (271)					
Omadacycline	2	16	70.5	13.3	
Tigecycline ^d^	1	4	86.7 ^d^	2.2 ^d^	
Minocycline	4	64	51.3	32.5	
Tetracycline	8	64	46.5	46.9	
Aztreonam	16	32	45.4	52.8	
Amikacin	4	64	86.7	12.5	
Ciprofloxacin	0.5	4	43.9	49.8	
Ceftazidime	4	64	52.8	42.1	
Ceftriaxone	64	64	38.7	59.8	
Imipenem	0.5	32	81.9	14.4	
Piperacillin-tazobactam	4	128	63.8	31.7	
*Acinetobacter baumannii* (198)					
Omadacycline	4	16			
Tigecycline	2	8			
Minocycline	8	16	45.5	28.3	
Tetracycline	64	64	16.2	81.3	
Amikacin	64	64	29.8	69.7	
Ciprofloxacin	4	4	21.2	76.8	
Cefepime	64	256	19.7	75.8	
Imipenem	32	64	24.7	75.3	
Piperacillin-tazobactam	128	128	23.2	75.3	
*Pseudomonas aeruginosa* (30)					
Omadacycline	32	32			
Tigecycline ^e^	8	16			
Minocycline	8	32			
Tetracycline ^e^	32	64	0.0	100.0 ^e^	
Amikacin	4	8	93.3	6.7	
Ciprofloxacin	0.25	4	73.3	23.3	
Cefepime	1	64	73.3	20.0	
Imipenem	1	16	80.0	20.0	
Piperacillin-tazobactam	8	128	63.3	20.0	

^a^Using FDA identified breakpoints for ABSSSIs

^b^Using FDA identified breakpoints for *Enterococcus faecalis*

^c^Using FDA identified breakpoints for *Klebsiella pneumoniae* and *Enterobacter cloacae*

^d^Using EUCAST clinical breakpoints for tigecycline

^e^Tigecycline and tetracycline are inherent resistant against *Pseudomonas aeruginosa* ccording to CLSI document M100ED29E

Omadacycline was highly active against enterococci (MIC_50/90_, 0.03/0.12 mg/L; 100.0% susceptible [ABSSSI breakpoints for *Enterococcus faecalis*]) isolates, whose resistance rates against tetracycline and minocycline were 76.0 and 52.0%, respectively (Tables 1 and 3). Against *E. faecalis*, MIC_50_ of omadacycline was 0.06 mg/L. Only one vancomycin-resistant *E. faecium* isolate was inhibited by 0.03 mg/L of omadacycline (Table 3).

The MIC_50/90_ values for all *S. pneumoniae* isolates, as well as for penicillin-resistant and tetracycline-resistant isolates, against omadacycline, were 0.06/0.06 mg/L (≥90.6% susceptible using CABP breakpoints). For tigecycline-non-susceptible isolates, the highest MIC value of omadacycline was 0.25 mg/L, though the susceptible rate was just 33.3%. Against viridans group streptococci and β-haemolytic streptococci, MIC_50/90_ values of omadacycline were 0.03/0.12 mg/L and 0.12/0.25 mg/L, respectively. About 86% of tetracycline-resistant *S. anginosus* group (85.7%), and 100.0% of *S. pyogenes*, were susceptible to omadacycline.

### Susceptibility of gram-negative isolates to omadacycline

The MIC distributions of Gram-negative bacilli isolates against omadacycline are shown in Tables 2 and 3. Omadacycline exhibited good in vitro activity against 651 *Enterobacterales* isolates studied (MIC_50/90_, 2/8 mg/L) inhibiting 81.7% of the isolates at ≤4 mg/L (ABSSSI susceptible breakpoint for *Enterobacter cloacae* and *K. pneumoniae*). All tigecycline-non-susceptible *Enterobacterales* isolates showed very high MICs against omadacycline (MIC_50/90_, 16/32 mg/L; Tables 2 and 3). Of note, omadacycline was significantly more active against *E. cloacae* (MIC_50/90_, 2/4 mg/L; 93.3% susceptible) and *Escherichia coli* (MIC_50/90_, 1/4 mg/L; 92.3% inhibited at ≤4 mg/L), than against *K. pneumoniae* (MIC_50/90_, 2/16 mg/L; 70.5% susceptible). Furthermore, omadacycline showed good activity against ceftazidime-non-susceptible and tetracycline-resistant *Enterobacterales* (MIC_50/90_, 2/16 mg/L for both; 76.4 and 72.6% inhibited at ≤4 mg/L, respectively), especially tetracycline-resistant *E. coli* (MIC_50/90_, 1/4 mg/L; 91.0% inhibited at ≤4 mg/L), ceftazidime-non-susceptible *E. cloacae* (MIC_50/90_, 2/4 mg/L; 93.8% susceptible), tetracycline-resistant *E. cloacae* (MIC_50/90_, 4/32 mg/L; 81.8% susceptible) and tetracycline-resistant *Citrobacter* spp. (MIC_50/90_, 2/16 mg/L; 81.8% inhibited at ≤4 mg/L). However, omadacycline showed a weaker activity against ceftazidime-non-susceptible (MIC > 4 mg/L) *K. pneumoniae* and *E. coli* isolates (62.5 and 86.8% inhibited at ≤4 mg/L, respectively) compared to ceftazidime-susceptible isolates (77.6 and 96.1% inhibited at ≤4 mg/L, respectively). Carbapenemase-producing/extended-spectrum β-lactamase (ESBL) phenotype positive *K. pneumoniae* isolates were less susceptible to omadacycline (72.3 and 57.6% susceptible at ≤4 mg/L, respectively) compared with ESBL phenotype negative isolates (84.0% susceptible at ≤4 mg/L, respectively).

When tested against non-fermenting bacteria, omadacycline demonstrated moderate activity against *Acinetobacter baumannii* (MIC_50/90_, 4/16 mg/L), and good activity against *Stenotrophomonas maltophilia* (MIC_50/90_, 2/4 mg/L), but very limited in vitro activity against *P. aeruginosa* (MIC_50/90_, 32/32 mg/L) isolates, which also exhibited low susceptibility to most antimicrobials tested (Tables 2 and 3). Furthermore, omadacycline was tested against *M. catarrhalis* isolates, with MIC_50/90_ values of 0.12/0.25 mg/L against this organism (Table 2).

## Discussion

Tigecycline has been an important option antibiotic for treatment of patients infected with tetracycline-resistant bacterial strains. Tigecycline, however, is available only as an intravenous formulation, while omadacycline is available both as an intravenous and oral formulation. Similar to other tetracyclines, omadacycline binds to 30S subunit of bacterial ribosome for inhibiting protein synthesis. Structure of omadacycline differs significantly from other tetracyclines and this compound represents a new aminomethylcycline subsclass [12]. Omadacycline overcomes the tetracycline efflux and ribosome protection mechanisms of bacterial resistance, due to modification at the C7 and C9 positions, respectively [13].

As the first agent of the aminomethylcycline class, omadacycline has a broad spectrum activity against Gram-positive and Gram-negative bacteria, and atypical pathogens including *Mycobacterium abscessus*, *Mycoplasmas*, *Ureaplasmas*, *Legionella* spp. and *Chlamydia* spp. [9, 14, 15]. Even against highly resistant bacterial isolates, such as MRSA, VRE, and PRSP, good activity has been observed for omadacycline [7, 10, 16].

Over the past decades, the incidence of MRSA has been slowly decreasing in the United States of America (USA), the United Kingdom, Australia and China [17–21]. However, it still remains a major issue for skin and skin structure infections [22, 23]. High omadacycline activity rates were observed against MRSA and tetracycline-resistant *S. aureus* isolates in the present study, with 96.9 and 97.4% susceptible rates, respectively, which is consistent with other reported data from USA and Europe [10]. In addition, the activity of omadacycline against α-hemolytic streptococci was impressive, especially against *S. pneumoniae*, and even PRSP and tigecycline-non-susceptible *S. pneumoniae* (100.0% susceptible).

Compared with data in the SENTRY antimicrobial surveillance program, MIC values of omadacycline against *Enterobacterales* isolates tested in this study, were similar (MIC_50/90_, 2/8 mg/L vs MIC_50/90_, 1 ~ 2/8 mg/L) [10, 16]. Omadacycline inhibited 81.7% of *Enterobacterales*, 92.3% of *E. coli* and 93.3% of *E. cloacae* isolates at ≤4 mg/L. Furthermore, omadacycline showed good activity against *E. coli* and *E. cloacae* isolates which were carbapenemase and ESBL phenotype positive, ceftazidime susceptible, or tetracycline susceptible (Table 2).

In China, the most common bacterial pathogen causing community acquired pneumonia (CAP) is *Mycoplasma pneumoniae*, accounting for 32.4% of 1500 children presenting with CAP in some studies [24, 25]. Furthermore, *P. aeruginosa*, *K. pneumoniae*, *E. coli* and *Acinetobacter* have been reported as significant CAP pathogens for hospitalized adults [24, 25]. According to guidelines of the Infectious Diseases Society of America and the American Thoracic Society, non-fermenting Gram-negative bacilli account for 19% (95% CI, 15–24%) of cases of hospital-acquired pneumonia (HAP), with *P. aeruginosa* implicated in 13.8% of HAP or ventilator-associated pneumonia (VAP) cases [26]. In the present study, omadacycline showed moderate in vitro activity against *K. pneumoniae* and *A. baumannii*, inhibiting 70.5 and 75.8% of the isolates at ≤4 mg/L, respectively (Table 2). It is possible that the *A. baumannii* and *K. pneumoniae* isolates tested in the present study may have originated from patients with HAP/VAP. And if that is the case, the higher MIC values of omadacycline seems reasonable. Compared with MIC_50/90_ values of minocycline (8/32 mg/L) and tigecycline (8/16 mg/L) against *P. aeruginosa*, MIC_50/90_ values of omadacycline (32/32 mg/L) were higher. Similar to its initial resistance to tetracycline and tigecycline [27], *P. aeruginosa* also exhibited low susceptibility to omadacycline.

This study has several limitations. The most obvious being the source of the isolates, as some strains were not isolated from patients with CAP or ABSSSIs. However, just like tigecycline is used in some non-approved clinical indications [28–30], we hope that the results of in vitro activity of omadacycline against strains isolated from the other infections can provide references for other clinical uses. Thus for a more comprehensive understanding of the in vitro antibacterial spectrum of omadacycline, we have also included other pathogens apart from those causing CAP and ABSSSIs, such as enterococci.

## Conclusions

In summary, omadacycline showed good in vitro activity against Gram-positive organisms and useful activity against most *Enterobacterales* strains isolated from China. In addition, omadacycline showed potent activity against several antimicrobial-resistant profile pathogens, including MRSA, PRSP, ESBL phenotype positive, and even carbapenemase-producing *E. coli*. Increased MIC values of omadacycline were observed in *K. pneumoniae* isolates in the present study, compared with other geographic areas recently studied [10, 16, 31, 32].

## Methods

### Organisms

A total of 1273 non-duplicate bacterial stains were collected from 25 teaching hospitals in China between January 2014 and December 2017. These isolates came from i) a multicenter surveillance program for CAP, HAP and ABSSSIs in China between 2014 and 2016; ii) a multicenter surveillance program for intra-abdominal infections (IAIs) and urinary tract infections (UTIs) in China from 2015 to 2016; iii) a surveillance program for infections caused by *Streptococcus* species in China in 2016; and iv) routine isolates causing respiratory tract infections (RTIs) and bloodstream infections (BSIs) in Peking Union Medical College Hospital from 2015 to 2017.

The 25 teaching hospitals are located in eighteen provinces across all the seven geographic regions in China. The strains studied were isolated from different clinical departments in each teaching hospital. According to sample type category, the majority was collected from respiratory tract (39.4%), including sputum (*n* = 435), broncho-alveolar lavage (*n* = 60), tracheobronchial aspiration (*n* = 5), and throat swab (*n* = 1). Other sources included urinary tract (21.5%), body fluid (19.2%), tissue (9.9%), abscess (6.9%), and others (3.2%). All the isolates were primarily identified at each local laboratory and confirmed in the central laboratory by Matrix-assisted laser desorption ionization time-of-flight mass spectrometer (bioMérieux, Vitek MS, USA) or 16S rDNA sequencing when necessary.

### Antimicrobial susceptibility testing

Susceptibility testing (omadacycline, tetracycline, minocycline and tigecycline) was performed by broth microdilution following Clinical and Laboratory Standards Institute (CLSI) documents M7-A9 [33]. Omadacycline powder was provided by Zai laboratory (Shanghai, China). The susceptibility testing of other antimicrobial agents was performed using commercial broth microdilution panels [Sensititre (ThermoFisher Diagnostics)]. *S. aureus* ATCC 29213, *E. faecalis* ATCC 29212, *S. pneumoniae* ATCC 49619, *E. coli* ATCC 25922, and *P. aeruginosa* ATCC 27853, were used as quality control strains. Results were interpreted in accordance with CLSI M100 guidelines [27]. Furthermore, the result interpretation of tigecycline was done in accordance with the U.S. FDA and European Committee on Antimicrobial Susceptibility Testing (EUCAST) 2019 guidelines [34]. Omadacycline clinical breakpoints for *S. aureus*, *S. pneumoniae*, *K. pneumoniae*, *E. cloacae* and *E. faecalis*, were released by the U.S. FDA on June 2018 [35]. We applied EUCAST clinical breakpoints for colistin (*Enterobacterales* excluding *Proteus* spp. and *Serratia* spp., S ≤ 2 mg/L, R > 2 mg/L; *P. aeruginosa*, S ≤ 4 mg/L, R > 4 mg/L) [34]. *E. coli*, *K. pneumoniae* and *Proteus mirabilis* isolates were screened for ESBL production using the disk diffusion method as per CLSI document M100-S29, screening criteria for potential ESBL production, i.e., ceftazidime (30 μg), cefotaxime (30 μg), with / without clavulanate (10 μg) [27]. Carbapenemase-producing *E. coli* and carbapenemase-producing *K. pneumoniae* were phenotypically resistant to carbapenems but the production of carbapenemase enzymes was not confirmed.

## Data Availability

All data generated or analysed during this study are included in this published article.
